# Accuracy and reliability of Manus, ChatGPT, and Claude in case-based dental diagnosis

**DOI:** 10.3389/froh.2025.1686090

**Published:** 2026-01-08

**Authors:** Ahmed A. Madfa, Abdullah F. Alshammari, Bassam A. Anazi, Yousef E. Alenezi, Khlood A. Alkurdi

**Affiliations:** 1 Department of Restorative Dental Science, College of Dentistry, University of Ha’il, Ha’il, Saudi Arabia; 2 Department of Basic Dental and Medical Science, College of Dentistry, University of Ha’il, Ha’il, Saudi Arabia; 3 Dental Research Centre, College of Dentistry, University of Ha’il, Ha’il, Saudi Arabia; 4 Ministry of Health, Qassim Health Cluster, King Saud Hospital, Unayzah, Saudi Arabia; 5 Institute of Dentistry, Queen Mary University of London, London, United Kingdom

**Keywords:** artificial intelligence, large language models, clinical decision-making, manus AI, ChatGPT, claude, intra-model consistency, dental education

## Abstract

**Introduction:**

Artificial intelligence (AI), particularly large language models (LLMs), is transforming healthcare education and clinical decision-making. While models like ChatGPT and Claude have demonstrated utility in medical contexts, their performance in dental diagnostics remains underexplored; additionally, the potential of emerging platforms, like Manus, is yet to be evaluated.

**Objective:**

To compare the diagnostic accuracy and consistency of the ChatGPT, Claude, and Manus—using authentic, case-based dental scenarios.

**Methods:**

A set of 117 multiple-choice questions based on validated clinical dental vignettes spanning various specialities was administered to each model under standardised conditions at two separate time points. Responses were scored against expert-validated answer keys. Inter-rater reliability was assessed using Cohen's kappa, and statistical comparisons were made using the chi-square, McNemar, and t-tests.

**Results:**

Claude and Manus consistently outperformed ChatGPT across both testing phases. In the second round, Claude and Manus achieved a diagnostic accuracy of 92.3%, compared to ChatGPT's 76.9%. Claude and Manus also demonstrated higher intra-model consistency (Cohen's kappa = 0.714 and 0.782, respectively) than ChatGPT (kappa = 0.560). Although the numerical trends favoured Claude and Manus, pairwise differences in accuracy did not reach statistical significance.

**Conclusion:**

Claude and Manus demonstrated numerically higher diagnostic performance and greater response stability compared with ChatGPT; however, these differences did not reach statistical significance and should therefore be interpreted cautiously. This variability across models highlights the need for larger-scale evaluations. These findings underscore the importance of considering both accuracy and consistency when selecting AI tools for integration into dental practice and curricula.

## Introduction

The emergence of artificial intelligence (AI) in healthcare is transforming the way clinicians, educators, students, and stakeholders approach patient care, education, and policymaking (1). AI-driven tools have rapidly advanced in areas such as diagnostic imaging, personalised treatment planning, and clinical decision-making, allowing healthcare workers to make faster, more accurate decisions. Furthermore, AI offers new avenues for research and innovation, reducing workloads and enhancing the quality of care delivered across diverse specialities (2). This revolution also holds promise in dentistry, where oral health professionals can leverage AI to improve both clinical practice and educational experiences (3). The increasing clinical, scientific, and educational uses of ChatGPT in dentistry are highlighted in a recent thorough narrative review by Puleio et al., highlighting its potential to help academic writing, patient communication, diagnostic reasoning, and simulation-based learning (4).

In the context of oral health, AI is increasingly being used to support diagnostics, interpret imaging, predict treatment outcomes, and guide students through simulated learning experiences. Dental students and faculty can benefit immensely from AI-powered platforms that provide rapid, evidence-based feedback, allowing for more interactive, self-directed learning (3). Practising dentists may also use AI tools as chairside aids to enhance diagnostic accuracy for complex cases, improving efficiency and ensuring that patients receive high-quality care. Moreover, for health stakeholders and policymakers, the incorporation of AI into oral healthcare holds potential for improving resource allocation, quality assurance, and continuous professional development. These advances align with a broader global trend toward digital transformation across health disciplines (5). ChatGPT and other LLM-based systems already show significant value across various domains, as noted by Puleio et al., underscoring the necessity of systematic assessments of their effectiveness in dental contexts (4).

Within this evolving landscape, large language models (LLMs), such as ChatGPT (developed by OpenAI), Claude (developed by Anthropic), and Manus (developed by the startup Monica), have demonstrated a unique capacity to understand complex text-based queries, retrieve knowledge from vast datasets, and produce nuanced, context-aware responses (4). ChatGPT, a transformer-based LLM, is trained on a broad corpus and excels at conversational responses; Claude emphasises safety and alignment with user needs and is known for its concise and carefully reasoned output; and Manus, though less publicised, has been designed with robust integrative algorithms that synthesise data and potentially enhance accuracy in highly specialised domains like healthcare (6–8). Taken together, these models may significantly contribute to knowledge dissemination and skill development in dentistry.

Several preliminary studies have examined the effectiveness of AI language models in dental contexts. ChatGPT has demonstrated the ability to correctly answer a substantial proportion of dental knowledge-based multiple-choice questions (MCQs), suggesting its potential as a valuable educational support tool (9). Claude has shown competence in generating evidence-based diagnostic suggestions for oral pathologies, highlighting its usefulness in clinical training environments (10). However, to the best of our knowledge, no prior studies have evaluated the effectiveness of Manus in dental contexts, and its potential to support clinical decision-making remains largely unexplored.

Despite their promising outcomes, the need for direct, standardised comparisons among these models is underscored by variations in diagnostic accuracy, reasoning depth, and consistency over time. Direct comparative research examining how these models perform under identical clinical conditions remains lacking, especially when applied to realistic, case-based dental questions. Differences in training data, model architecture, and versioning may result in substantial variations in clinical utility. Thus, a direct, standardised comparison would generate valuable insight into which model most reliably supports dental professionals' and students' diagnostic processes.

Authentic case vignettes (structured to mimic actual patient presentations) offer an ideal means of assessing diagnostic reasoning and decision-making capabilities. Unlike purely theoretical or factual questions, case vignettes require the integration of diverse data points, including demographic information, chief complaints, history, clinical findings, and diagnostic imaging (11). This approach better simulates the complexity and nuance of real-world practice, allowing for a more robust evaluation of an AI model's capacity to provide accurate and clinically sound guidance. Furthermore, testing these models across different time points enables an assessment of consistency and knowledge retention, further approximating clinical reality, where stable performance over time is paramount.

The primary aim of this research was to evaluate and compare the diagnostic reasoning and clinical decision-making performance of the three AI models described above, ultimately providing evidence to inform their future integration into oral healthcare education and practice. Using a PICO framework, the present study aimed to address the following research question: “How do ChatGPT, Claude, and Manus compare in terms of diagnostic accuracy and consistency when responding to authentic clinical dental scenarios across two time points?”.

## Methodology

### Ethical considerations

The study did not include human participants or involve the use of identifiable personal data; therefore, informed consent was not required. Nonetheless, the research protocol was reviewed and approved by the Medical Ethical Committee of the College of Dentistry, University of Ha'il, Saudi Arabia (Approval No. H-2025-619). All methodological steps complied with established ethical principles for AI-related research, ensuring transparency, fairness, and adherence to academic integrity.

### Study design

A structured, comparative, longitudinal study design was employed to evaluate the diagnostic reasoning and clinical decision-making abilities of three LLMs: ChatGPT (GPT-4-turbo, OpenAI, version released January 2024), Claude (Claude 3 Opus, Anthropic, version released March 2024), and Manus (latest version as of March 2025). The objective was to assess baseline accuracy and temporal consistency by testing the models on the same questions at two different time points.

### Question development and validation

A total of 117 MCQs were constructed based on authentic clinical vignettes across all major dental specialities, including Oral and Maxillofacial Radiology, Oral Medicine and Oral Pathology, Periodontology, Endodontics, Operative and Restorative Dentistry, Prosthodontics, Paediatric Dentistry, and Orthodontics (Supplementary Material S1).

Each MCQ included patient demographics, chief complaints, history, clinical findings, diagnostic imaging findings, and any additional test results as required for accurate clinical decision-making.

All questions followed a single-best-answer format with 4 options. Correct answers were derived based on current, evidence-based clinical guidelines, such as those of the American Dental Association, European Federation of Periodontology, European Academy of Paediatric Dentistry, and American Association of Endodontists.

A panel of four experienced dental consultants (with ≥ 7 years of clinical and teaching experience) reviewed all questions and answer keys to ensure content validity and clinical relevance.

Each consultant independently reviewed all items, and any discrepancies in answer keys or question clarity were resolved through a consensus meeting. Questions were iteratively revised until full agreement was reached among all experts. To ensure adequate content coverage, consultants evaluated items both within and outside their respective specialties, confirming balanced representation across major dental domains. Although a formal inter-rater reliability coefficient was not calculated due to the consensus-based approach, unanimous agreement was required before finalizing each question and answer key.

### AI interaction and data collection

In the initial evaluation phase (Phase 1), each of the 117 MCQs (12–14) was individually submitted to the interface of each AI model under identical, standardised conditions (Supplementary Material S1). To ensure fairness and reproducibility, identical prompts and phrasing were used across all models without providing any additional guidance, hints, or background context (Supplementary Material S2). The full responses generated by each AI model were then carefully documented as received, without modification, in a structured Microsoft Excel file. For each interaction, metadata, including the model's name and version, the query date, the question ID, the selected answer, and the model's complete textual explanation, were recorded for subsequent analysis.

Ten days after the first round of testing, the same 117 questions were re-administered to each AI model under the same strict protocol (Phase 2). This follow-up evaluation was conducted to assess whether the models' diagnostic reasoning and answer accuracy remained consistent over time, as well as to identify any variations in their responses or apparent knowledge retention. Again, all outputs were captured verbatim and logged in the data sheet alongside the corresponding metadata for comparison with the initial results.

### Scoring and response evaluation

Two independent reviewers, both dental undergraduates, classified all responses as correct or incorrect by comparing them to the pre-validated answer keys. Undergraduate reviewers were selected because the task required only binary scoring of responses against consultant-validated answer keys, rather than clinical judgement or interpretation. The reviewers had completed the relevant clinical courses and were trained to ensure scoring accuracy and consistency. Inter-rater reliability was calculated using Cohen's kappa, with a target *κ* ≥ 0.80 indicating strong agreement.

Where provided, textual justifications were noted separately for subsequent qualitative analyses of clinical reasoning, although accuracy scoring was based solely on the selected multiple-choice option.

### Data management and statistical analysis

All responses from both phases were systematically compiled into a structured Microsoft Excel file, with each row representing one question–model interaction. Relevant metadata, including the model's name and version, question ID, test phase, selected answer, correctness classification, and any explanatory text provided, were recorded. Prior to analysis, the data were cleaned and checked for completeness, and scoring accuracy was verified independently by two reviewers. Inter-rater reliability was calculated using Cohen's kappa, with *κ* ≥ 0.85 indicating strong agreement.

The finalised dataset was then exported to IBM SPSS Statistics (Version 28) for statistical analysis. Descriptive statistics, including mean accuracy scores, standard deviations, and frequency distributions, were calculated separately for each model across both time points. Differences in accuracy between models were assessed using chi-square tests, and McNemar's tests were performed to evaluate changes in performance across phases. Independent-samples t-tests were employed to explore variations in mean accuracy across dental specialities. Statistical significance was set at *p* < 0.05.

## Results

### Overall diagnostic accuracy (first and second measurements)

In the first measurement, Claude demonstrated the highest diagnostic accuracy, answering 107 out of 117 questions correctly (91.5%), followed closely by Manus with 106 correct responses (90.6%). ChatGPT exhibited comparatively poorer performance, correctly answering 87 questions (74.4%). Correspondingly, the number of incorrect answers showed the inverse, with Claude and Manus giving 10 (8.5%) and 11 (9.4%) incorrect responses, respectively, while ChatGPT provided 30 incorrect responses (25.6%); see Table 1.

**Table 1:** Diagnostic accuracy of AI models in two measurements.

First measurement			
Variables	Manus	Chat GPT	Claude
Correct answer	106 (90.6%)	87 (74.4%)	107 (91.5%)
Incorrect answer	11 (9.4%)	30 (25.6%)	10 (8.5%)
Second measurement			
Variables	Manus	Chat GPT	Claude
Correct answer	108 (92.3%)	90 (76.9%)	108 (92.3%)
Incorrect answer	9 (7.7%)	27 (23.1%)	9 (7.7%)

**Figure 1:**
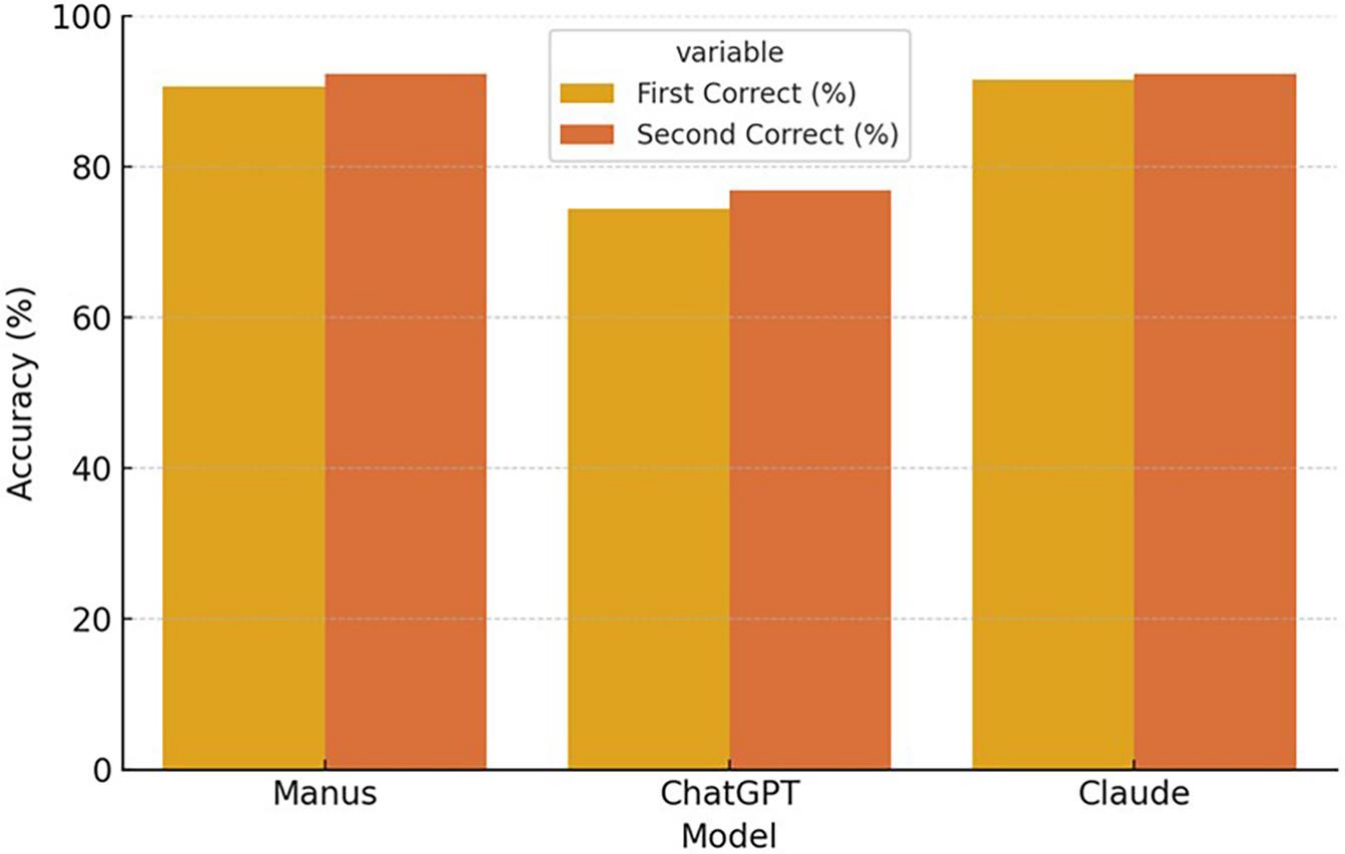
Diagnostic accuracy across two time points.

In the second measurement, both Manus and Claude achieved the same performance, each correctly answering 108 questions (92.3%), while ChatGPT's performance improved slightly, with 90 correct responses (76.9%). The number of incorrect answers was reduced to nine (7.7%) for both Manus and Claude and 27 (23.1%) for ChatGPT. Figure 1 compares the diagnostic accuracy of Manus, ChatGPT, and Claude during the first and second rounds of testing.

### Consistency over time (agreement between first and second measurements)

To evaluate intra-model consistency, responses from the first and second measurements were cross-tabulated, and Cohen's kappa coefficient was calculated to quantify agreement levels. Manus showed high consistency between the two time points. Of the 106 initially correct responses, 105 (89.7%) remained correct in the second measurement, with only one response changing to incorrect (0.9%). Among the 11 initially incorrect responses, three became correct, and eight remained incorrect. The Cohen's kappa coefficient for Manus was 0.782, indicating substantial agreement (Table 2).

**Table 2:** Intra-model consistency between first and second measurements.

Manus			
Measurement		Second Measurement	
		Correct	Incorrect
First measurement	Correct	105 (89.7%)	1 (0.9%)
	Incorrect	3 (2.6%)	8 (6.8%)
Measure of Agreement Kappa	.782		
ChatGPT			
Measurement		Second Measurement	
		Correct	Incorrect
First measurement	Correct	79 (67.5%)	8 (6.8%)
	Incorrect	11 (9.4%)	19 (16.2%)
Measure of Agreement Kappa	.560		
Claude			
Measurement		Second Measurement	
		Correct	Incorrect
First measurement	Correct	105 (89.7%)	2 (1.6%)
	Incorrect	3 (2.6%)	7 (6.0%)
Measure of Agreement Kappa	.714		

**Figure 2:**
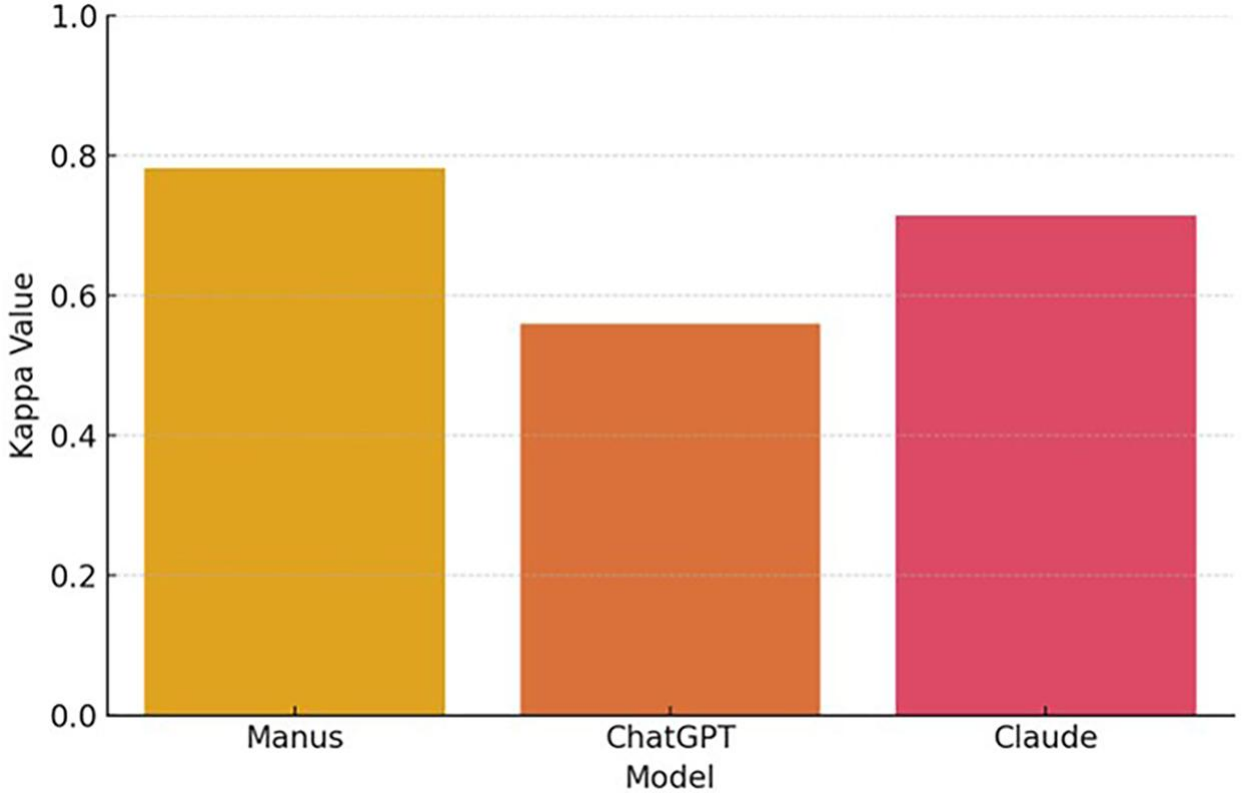
Inter-Measurement agreement (Cohen's Kappa).

Claude demonstrated similar stability. Of the 107 initially correct responses, 105 (89.7%) remained so, while two turned incorrect in the second round (1.6%). Of the 10 initially incorrect responses, three became correct, while seven remained incorrect. The kappa coefficient for Claude was 0.714, also reflecting substantial agreement. ChatGPT, in contrast, showed lower stability. Of the 87 correct responses in the first measurement, 79 (67.5%) were retained in the second, while eight turned incorrect (6.8%). Among the 30 initially incorrect responses, 11 were corrected and 19 remained incorrect. ChatGPT's kappa coefficient was 0.560, indicating moderate agreement and suggesting more variability in its responses across time. Figure 2 illustrates the consistency of each model between the two testing phases, as measured by Cohen's kappa.

### Cross-model comparisons of accuracy

Pairwise comparisons between models were conducted to further examine differences in performance and their statistical significance. Comparing Manus to ChatGPT, Manus consistently outperformed ChatGPT in both measurements. In the second round, 85 of ChatGPT's correct responses matched those of Manus, while Manus provided only two incorrect responses where ChatGPT was correct. However, the difference in the correct response rate between the two was not statistically significant (*p* = 0.273), possibly due to the relatively small number of questions (Table 3).

**Table 3:** Cross-comparison of accuracy between three AI language models, Manus, ChatGPT and Claude.

AI Models	Answers				*P* Value
	Both A&B correct	A correct & B incorrect	A incorrect & B correct	Both A&B incorrect	
Manus (A) vs. ChatGPT (B)	85 (78.7%)	23 (19.7%)	2 (1.7%)	7 (6.0%)	.273
Manus (A) vs. Claude (B)	105 (89.7%)	7 (6.0%)	2 (1.7%)	3 (2.6%)	.714
ChatGPT (A) vs. Claude (B)	88 (82.2%)	2 (1.7%)	19 (17.8%)	8 (6.8%)	.352

In comparisons between Claude and ChatGPT, Claude showed higher accuracy. Of ChatGPT's 90 correct responses in the second round, 88 matched Claude's correct responses, while only two questions were answered correctly by ChatGPT but not by Claude. The difference between the two models was not statistically significant (*p* = 0.352), although Claude's overall accuracy and consistency trends were superior.

In contrast, Claude and Manus showed near-identical performance in the second measurement. Claude correctly answered 105 of the questions that Manus also answered correctly, with only minimal divergence between them (two and three incorrect responses, respectively). The *p*-value for this comparison was 0.714, supporting the absence of a statistically significant difference in diagnostic accuracy between the two models.

## Discussion

The present study evaluated the diagnostic accuracy of three LLMs, Claude, Manus, and ChatGPT, in responding to dental examination questions. The results indicate that Claude and Manus consistently outperformed ChatGPT, reflecting both stronger reasoning mechanisms and potential domain-specific fine-tuning. These findings are consistent with prior evaluations of LLMs in medical and dental contexts (12–24). For example, ChatGPT (GPT-4) achieved 86.6% on the USMLE (21) and 88.6% on dental board-style questions (22), demonstrating strong general medical knowledge, yet it showed limitations in specialized dental domains compared with Claude and Manus. The superior performance of Claude and Manus may relate to their model architecture, structured clinical training datasets, or fine-tuning with exam-style questions, although the precise details of their training corpora remain proprietary (23, 24).

The differences in performance were most evident in the frequency of incorrect responses. Across both assessment rounds, ChatGPT produced nearly three times more errors than Claude or Manus, highlighting a notable discrepancy in reliability. This aligns with prior observations, such as those by Rao et al. (25), which noted that ChatGPT may present highly confident yet incorrect responses in clinical scenarios. ChatGPT exhibited a modest improvement in the second round (from 74.4% to 76.9%), suggesting some adaptability to question structures or increased familiarity with prompt formats, though this requires further evaluation. In contrast, Claude and Manus maintained relatively stable performance, with error rates ranging from 7.7%–9.4% across rounds, indicating more consistent accuracy.

Intra-model consistency was assessed using Cohen's kappa coefficient. Manus demonstrated the highest stability, retaining 89.7% of initially correct responses (*κ* = 0.782), followed by Claude (86.1%, *κ* = 0.714). ChatGPT showed moderate agreement (*κ* = 0.560), retaining only 67.5% of correct responses. These findings are consistent with prior studies reporting variability in repeated outputs from GPT-based models, particularly in complex or non-deterministic scenarios (26, 27). The higher stability of Claude and Manus may result from differences in architecture or targeted fine-tuning on domain-specific tasks, reducing stochastic output patterns. Minor fluctuations were observed even in these models (1.6%–1.9% of responses), indicating that small variations can occur despite high overall performance.

Pairwise comparisons were conducted to assess relative performance among the models. Although Claude and Manus consistently outperformed ChatGPT, the differences did not reach statistical significance, likely due to the modest sample size of test items. Manus and ChatGPT shared 85 correct responses in the second round, with ChatGPT outperforming Manus in only two instances. Claude and Manus exhibited nearly identical performance, correctly answering 105 of the same questions, with only minimal divergence (two questions unique to Claude and three unique to Manus; *p* = 0.714). These results suggest that both Claude and Manus achieved comparable proficiency in structured diagnostic tasks, supporting prior reports of parity or superiority of domain-optimized LLMs over general-purpose models in medical reasoning contexts (23, 24, 28, 29).

The intra-model stability also revealed patterns in how responses changed between assessment rounds. ChatGPT corrected only 11 of 30 initial errors while changing eight previously correct answers to incorrect, demonstrating greater variability than the other models. Claude and Manus showed far fewer such changes, further reflecting their more deterministic or fine-tuned response mechanisms. These observations are consistent with prior benchmarking studies and internal documentation suggesting that targeted fine-tuning and more deterministic architectures contribute to reduced output fluctuation (23, 24).

Overall, the study highlights consistent trends in performance and stability among the three LLMs. Claude and Manus consistently demonstrated higher accuracy, lower error rates, and greater intra-model agreement than ChatGPT, which exhibited moderate reliability and a higher incidence of incorrect responses. Pairwise analyses reinforce that the performance of Claude and Manus is closely aligned, while ChatGPT exhibits variability both in accuracy and response consistency across repeated assessments. These patterns are consistent with prior research examining large language models in medical and dental educational settings, reflecting the influence of model architecture, training data, and task-specific optimization on performance outcomes (21–31).

The lack of statistical significance in all pairwise comparisons underscores a key study limitation. The relatively small number of test questions (*n* = 117) may have reduced the power to detect meaningful differences. Although Manus and Claude demonstrated higher accuracy and consistency than ChatGPT across both testing phases, these differences were not statistically significant. As such, the observed performance trends should be interpreted as preliminary rather than conclusive. As such, while the observed numerical trends favour Claude and Manus over ChatGPT, larger-scale evaluations are warranted to substantiate these preliminary observations. Importantly, the convergence of Claude and Manus in diagnostic accuracy suggests a promising future for domain-specific LLMs tailored for healthcare. While ChatGPT performs respectably, it may be more prone to minor lapses in domain fidelity or contextual interpretation, particularly regarding specialised medical or dental knowledge.

### Clinical implications

The findings of this study have several important clinical and educational implications. First, the high diagnostic accuracy and intra-model consistency demonstrated by Claude and Manus suggest that these AI models may serve as reliable adjunctive tools in dental education and clinical decision-making. Their consistent performance across repeated assessments indicates potential for use in formative assessments and board exam preparation and as real-time clinical support systems, particularly in settings with limited access to specialists. Second, while ChatGPT showed moderate accuracy and lower stability, it nevertheless performed at a level that could support general dental learning and preliminary case reviews. However, due to its greater response variability, it should be used with caution in contexts requiring high reliability. Third, the similarity in performance between Claude and Manus underscores that domain-specific fine-tuning and model design can substantially enhance clinical utility. The integration of such models into digital dental platforms, patient education materials, and case-based simulations could streamline diagnostic workflows and improve training outcomes for dental professionals. Finally, although none of the models should be relied upon as standalone diagnostic tools, their potential to support differential diagnosis, reinforce learning, and reduce clinician cognitive load makes them promising components in the future of AI-augmented dental care. Looking ahead, future developments of ChatGPT and similar LLMs may significantly expand their role in dentistry. As multimodal capabilities advance, these models are expected to integrate radiographic images, clinical photographs, and 3D scans to support more comprehensive diagnostic reasoning. Improved domain-specific fine-tuning, alignment with dental clinical guidelines, and incorporation of safety-focused guardrails may enhance accuracy and reliability in real patient scenarios. Additionally, integration with electronic dental records, personalised treatment planning systems, and chairside decision-support tools could position ChatGPT as an interactive educational and clinical companion. As these technologies evolve, establishing robust validation frameworks and regulatory standards will be essential to ensure safe, ethical, and effective deployment within dental education and practice.

This study has several limitations, including a small sample size and the exclusive use of MCQs, which may restrict generalisability and fail to capture the models' broader reasoning abilities. The static testing format also overlooks interactive elements crucial in clinical settings. Additionally, the questions may not fully represent all dental specialities or complexity levels, limiting applicability to broader educational and clinical contexts, as model performance might vary with tailored datasets or prompts. Lastly, the study did not assess the quality of the answer rationales, and the results reflect a single time point, highlighting the need for ongoing evaluations to track model development over time. Although a qualitative analysis of the models' reasoning was not conducted, the recorded justifications provide a foundation for future studies to explore AI reasoning and performance differences in depth. Additionally, the exclusive reliance on MCQs represents a limitation. MCQs were chosen because they offer a standardized, objective, and reproducible method for comparing accuracy across models and reflect the structure of most dental board examinations. However, this format does not fully capture multi-step clinical reasoning or the depth of diagnostic justification. Future research should incorporate open-ended clinical scenarios, interactive case simulations, or multimodal inputs to more comprehensively assess LLM reasoning.

## Conclusions

This study compared the diagnostic accuracy and consistency of Manus, Claude, and ChatGPT in responding to case-based dental questions. Across both assessment rounds, Manus and Claude showed numerically higher accuracy and greater response stability compared with ChatGPT; however, these differences did not reach statistical significance and should therefore be interpreted cautiously. The observed trends indicate the potential of these models as supportive tools in dental education and clinical reasoning, but further research with larger datasets and more diverse assessment formats is required to confirm these preliminary findings. As LLMs continue to evolve, their integration into dental practice and education should be guided by careful evaluation of both accuracy and consistency.

## Data Availability

The datasets presented in this study can be found in online repositories. The names of the repository/repositories and accession number(s) can be found in the article/Supplementary Material.
