# Supplementary material for: Accuracy and reliability of Manus, ChatGPT, and Claude in case-based dental diagnosis
Source: Front Oral Health. 2026 Jan 8;6:1686090. doi: 10.3389/froh.2025.1686090 (PMC12823890; doi:10.3389/froh.2025.1686090)
Supplement: Supplementary file 1 [file Table2.docx]

## **S1.1 System-Level Instructions**

**System Instruction applied to all AI models:**

“You are an AI model participating in a controlled evaluation setting. For each question, you must select *only one* final answer from the options provided (A, B, C, or D). You are strictly prohibited from providing explanations, reasoning steps, justifications, or any additional commentary. Do not discuss alternative options, related concepts, or contextual information. Your output must consist solely of a single letter (A, B, C, or D). Use only the information contained within the question. No external knowledge, assumptions, or inferred information beyond the given MCQ item is allowed.”

## **S1.2 Exact Prompt Used for Every MCQ Item**

**Prompt Template (identical for all questions):**

“Select the correct answer from the following multiple-choice options (A, B, C, or D). Provide **only the single letter**corresponding to your final answer. **Do not provide any explanation.**

Question:
[Insert MCQ item here]”

**Example Format:**

**Prompt used for Question #1:**
“Select the correct answer from the following multiple-choice options (A, B, C, or D). Provide only the single letter corresponding to your final answer. Do not provide any explanation.
Question:
[Full text of MCQ #1]”

**Prompt used for Question #2:**
(same template; only the question text changes)

## **S1.3 Constrained Interaction Protocol**

“All AI models were evaluated under a standardized and constrained interaction protocol aligned with the NIST Artificial Intelligence Risk Management Framework (AI RMF 1.0, 2023). The protocol restricted model behavior to ensure reproducibility, consistency, and controlled output generation.

Each model was required to:

1. Follow a fixed system instruction prohibiting explanations, reasoning, or interpretive content.
2. Respond exclusively with a single letter (A, B, C, or D).
3. Use only the information presented within the MCQ prompt.
4. Avoid external knowledge, assumptions, or inference beyond the question stem.
5. Receive identical, pre-specified prompts for all MCQ items.

This controlled evaluation environment adhered to NIST RMF recommendations for pre-specified interaction conditions and constrained output formats necessary for reproducible AI assessment.”

# **References**

1. **National Institute of Standards and Technology (NIST).** Artificial Intelligence Risk Management Framework (AI RMF 1.0). Gaithersburg, MD: U.S. Department of Commerce; 2023.
2. **Liu X, Cruz Rivera S, Moher D, Calvert MJ, Denniston AK.** Reporting guidelines for clinical trials evaluating artificial intelligence interventions: the SPIRIT-AI and CONSORT-AI guidelines. *Lancet Digit Health.*2020;2(10):e537-e538.
3. **International Organization for Standardization (ISO).** ISO/IEC 24029-1:2021 — Artificial Intelligence (AI) — Assessment of the robustness of neural networks — Part 1: Overview. Geneva: ISO; 2021.
